# Beyond reward prediction errors: the role of dopamine in movement kinematics

**DOI:** 10.3389/fnint.2015.00039

**Published:** 2015-05-27

**Authors:** Joseph W. Barter, Suellen Li, Dongye Lu, Ryan A. Bartholomew, Mark A. Rossi, Charles T. Shoemaker, Daniel Salas-Meza, Erin Gaidis, Henry H. Yin

**Affiliations:** Department of Psychology and Neuroscience, Department of Neurobiology, Center for Cognitive Neuroscience, Duke UniversityDurham, NC, USA

**Keywords:** dopamine, substantia nigra, basal ganglia, movement, reward prediction error, striatum

## Abstract

We recorded activity of dopamine (DA) neurons in the substantia nigra pars compacta in unrestrained mice while monitoring their movements with video tracking. Our approach allows an unbiased examination of the continuous relationship between single unit activity and behavior. Although DA neurons show characteristic burst firing following cue or reward presentation, as previously reported, their activity can be explained by the representation of actual movement kinematics. Unlike neighboring pars reticulata GABAergic output neurons, which can represent vector components of position, DA neurons represent vector components of velocity or acceleration. We found neurons related to movements in four directions—up, down, left, right. For horizontal movements, there is significant lateralization of neurons: the left nigra contains more rightward neurons, whereas the right nigra contains more leftward neurons. The relationship between DA activity and movement kinematics was found on both appetitive trials using sucrose and aversive trials using air puff, showing that these neurons belong to a velocity control circuit that can be used for any number of purposes, whether to seek reward or to avoid harm. In support of this conclusion, mimicry of the phasic activation of DA neurons with selective optogenetic stimulation could also generate movements. Contrary to the popular hypothesis that DA neurons encode reward prediction errors, our results suggest that nigrostriatal DA plays an essential role in controlling the kinematics of voluntary movements. We hypothesize that DA signaling implements gain adjustment for adaptive transition control, and describe a new model of the basal ganglia (BG) in which DA functions to adjust the gain of the transition controller. This model has significant implications for our understanding of movement disorders implicating DA and the BG.

## Introduction

The role of dopamine (DA) in behavior has remained controversial despite decades of research (Cannon and Palmiter, 2003; Cagniard et al., 2006; Jin and Costa, 2010; Leblois et al., 2010; Rossi et al., 2013a). Degeneration of DA neurons results in Parkinson's disease, associated with severe motor deficits, suggesting that dopaminergic signaling is essential for movement. Some studies showed that DA activity is correlated with the initiation and termination of instrumental actions, locomotion, and postural adjustments (Jin and Costa, 2010; Wang and Tsien, 2011a; Fan et al., 2012; Barter et al., 2014). Others, however, found no clear relationship between DA activity and movement (Schultz et al., 1983; Romo and Schultz, 1990), and concluded instead that phasic DA signal encodes reward prediction error, the difference between the expected reward and the actual reward, which can serve as a teaching signal in reinforcement learning (Schultz et al., 1997).

One limitation of previous work is that detailed movement parameters were rarely measured continuously and quantified. To obtain continuous measures of behavioral parameters, we recently began to combine video tracking and wireless *in vivo* recording, to study the relationship between movement kinematics and activity in the BG. We found that medium spiny projection neurons in the sensorimotor striatum, a major target of DA projections, showed activity reflecting the horizontal and vertical vector components of movement velocity (Kim et al., 2014). On the other hand, the projection neurons from the substantia nigra pars reticulata (SNr), a major output nucleus of the BG that receives direct striatal input, showed activity reflecting distinct components of position vectors (Barter et al., 2015). These findings support a new model of BG function, according to which these nuclei collectively function as a transition control system situated in a hierarchy of negative feedback control systems that vary outputs to reach inputs specified by their reference signals. In the sensorimotor circuit, the relevant proprioceptive transition represents the rate of change in the representation of body configurations, so that movement velocity can be a key controlled variable. The nigral output neurons, in turn, can integrate the striatal velocity signals to generate commands representing desired position, which are then used to alter the reference signals for downstream position controllers for body orientation and configuration in the midbrain and diencephalon (Yin, 2014b).

What is the role of DA in this circuit? There are massive nigrostriatal DA projections which synapse on the neck of the dendritic spines, the sites of glutamatergic corticostrial projections. We hypothesize that DA serves as gain modulation for the movement velocity controller in the BG, e.g., via the corticostriatal projections (Yin, 2014b). Together with glutamatergic inputs DA signals can determine the magnitude of the descending signal from the velocity controller, and the *rate of change* in BG outputs (Kim et al., 2014; Barter et al., 2015). This hypothesis predicts that the firing rate of DA neurons is also related to movement velocity.

To test this hypothesis, we simultaneously measured movement kinematics as well as single unit activity from DA neurons in the substantia nigra pars compacta (SNc) in unrestrained mice (Fan et al., 2011). As predicted, we found that the phasic activity of DA neurons reflected distinct vector components of movement velocity and acceleration. Using a transgenic mouse line in which channelrhodopsin 2 is expressed selectively in DA neurons, we found that photo-stimulation of DA neurons in the SNc could also generate movements reliably.

## Methods

### Subjects

Eleven male C57BL6/J mice (25–35 g) were used in the electrophysiology experiments. Seven mice (two males, and five females) were used in the optogenetics experiments. All procedures were approved by the Duke University Institutional Animal Care and Use Committee. To make mice perform movements repeatedly, we gave them limited access to water. After the recording session each day, they had free access to water for 1 h. Each mouse received about 0.5 to 1 ml of 10% sucrose during the experimental session. When they had free access to water afterwards, they consumed ~2 ml. The health of all mice was monitored daily.

### Behavior

**Figure 1:**
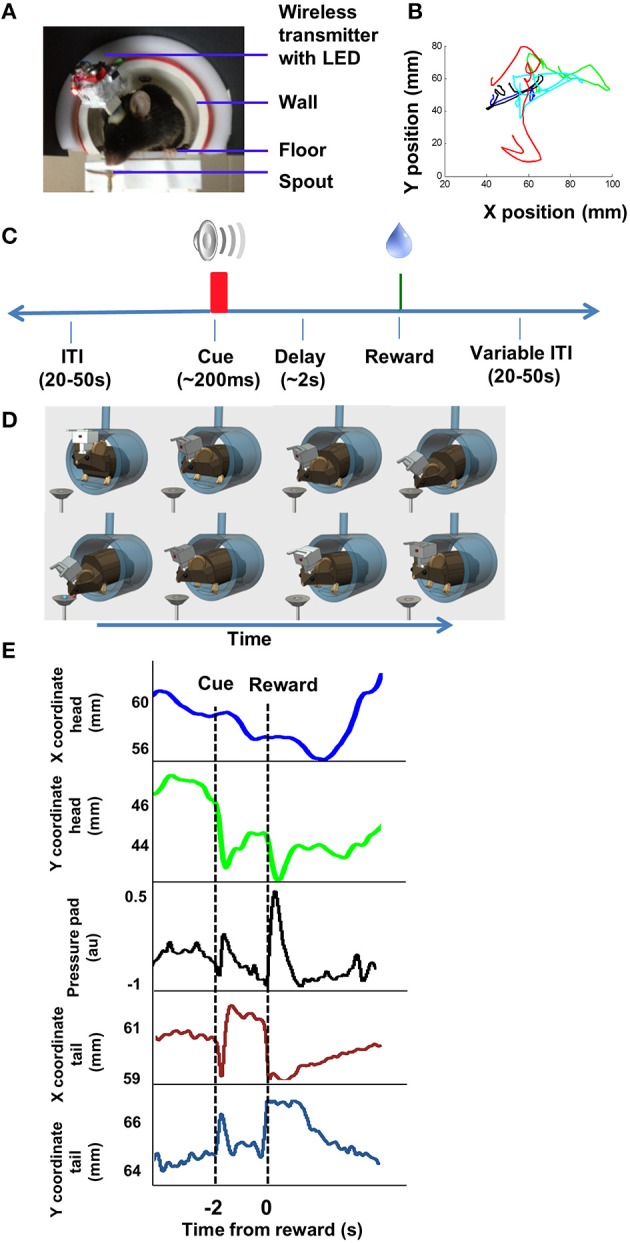
**Behavior and video tracking in unrestrained mice. (A)** Mice perched on an elevated platform housed in a tube, wearing a miniaturized 16 channel wireless headstage (~3.8 g). The camera (not shown) is facing the animal. **(B)** Illustration of movement trajectory. The mouse starts to move following presentation of the cue (CS), and moves again following the presentation of the reward (US, 13 μl 20% sucrose solution). Each color illustrates the path on a single trial, showing variability from trial to trial. **(C)** Illustration of the Pavlovian trace conditioning task used (Barter et al., 2015). **(D)** Cartoon illustration of the movements. Top row illustrates movement toward the spout; bottom row illustrates movement back to the starting position. **(E)** Illustration of movement tracked by the head LED. Pressure pads were placed underneath the animal, so that changes in pressure exerted by the hind paws can be measured. Pressure pad measures as well as video tracking of the tail demonstrate that the movements were not restricted to the head. Position coordinates are mm from frame edge.

We used a simple Pavlovian trace conditioning task to study the phasic DA responses in relation to rewards (unconditional stimulus, US) and cues predicting rewards (conditional stimulus, CS). In this task, the mouse stands on an elevated platform (4 × 5 cm, elevated 40 cm) and its movement can be monitored with a camera facing it at 30 frames/s (Figure 1). An auditory cue predicted the delivery of a sucrose reward, and the mouse reliably moved following the cue and following the reward. The experimental apparatus and procedures are the same as what we used in two recent studies (Kim et al., 2014; Barter et al., 2015). This design allows us to record from DA neurons while monitoring movement kinematics, using a small LED light positioned on the headstage. It also minimizes z-axis movements.

Each trial began with the presentation of a tone (100 ms, 4 kHz, 21.6 dB) followed by the delivery of 13 μl 10% sucrose solution dispensed by a Valvelink 8.2 (AutoMate Scientific) and delivered through a spout fixed to the platform. The sucrose solution was delivered ~2 s after the termination of the tone. Each session contained 50–150 trials, with a variable inter-trial-interval of 20–50 s. Each session lasted approximately 1 h.

For sucrose/air puff sessions, either an ABAB design was used, in which reward trials and air puff trials were presented in blocks, or a AB design was used, in which sucrose trials were followed by air puff trials. The same auditory cue was used. Air puffs were 200 ms in duration and delivered from a computer-controlled 1500 series dispenser (EFD, 12 PSI).

### Neural recording and data analysis

Sixteen-channel electrode arrays (Innovative Neurophysiology) were lowered at the following stereotaxic coordinates in relation to bregma: 2.9–3 mm posterior, 1.2 mm lateral, and 4.6 mm below brain surface. Six mice were implanted in left nigra and the other six were implanted in the right nigra. The arrays consisted of 16 tungsten wires, 35 μm in diameter and 7 mm in length, arranged in a four by four configuration, attached to an Omnetics connector. Row spacing was 200 μm and electrode spacing was 150 μm. Electrode arrays were fixed to the skull with dental acrylic. Following the completion of the experiments, all mice were perfused and their brains sliced with a Vibratome and examined under a microscope to verify electrode placement.

The behavioral and electrophysiological data were recorded with a Cerebus data acquisition system (Blackrock) and analyzed with Matlab, Neuroexplorer, and Graphpad Prism. Single unit activity was recorded with miniaturized wireless headstages (Triangle BioSystems International), as described previously (Fan et al., 2011). Attached to the front end are miniature LEDs (2 mm, Osram). Single units were selected using online sorting algorithms and then re-sorted offline (Fan et al., 2011; Rossi et al., 2013a). Only single-unit activity with a clear separation from noise (at least five to one compared to the noise band) was used for data analysis.

### Kinematic variables

Position, velocity, and acceleration are vector quantities with both magnitude and direction. For movement measured with 2D video tracking, this vector has two components (x and y). X and Y head position vectors were differentiated to get X and Y velocity, and the second derivative was taken to obtain acceleration. X and Y velocity and X and Y acceleration were then split into positive and negative components to yield a total of eight kinematic variables: up and down velocity and acceleration, left and right velocity and acceleration.

We then compared the neural activity to kinematic variables. To assess the correlation between neural activity between firing rate and kinematics, we analyzed data from the entire session. A complete record of neural activity and the continuous kinematic variables for each session was analyzed in Matlab with a bin size of 30 ms. The analysis consisted of two steps: first, for each session, cross-correlation was performed between the firing rate of each neuron and each of the eight kinematic variables to determine the shift required for the highest correlation between the two signals. Second, the neural signal and the kinematic signal were shifted accordingly and a Pearson correlation was then performed to determine the correlation between the two signals. Classification of different functional classes of neurons was determined by the strength of the correlation between the kinematic variable and neural activity (*p* < 0.05).

### Optogenetic stimulation

By crossing *Th-cre* mice, which express Cre recombinase in tyrosine hydroxylase positive neurons, with a knockin line (Ai32, ChR2-EYFP) for Cre-dependent expression of channelrhodopsin 2 (Madisen et al., 2012), we generated *Th::Ai32* mice for selective activation of DA neurons.

Custom-made optic fibers (5 mm length below ferrule, 105-μm core diameter, 1.25-mm-OD ceramic zirconia ferrule; Precision Fiber Products) were lowered into the brain and secured in place with dental acrylic and skull screws (Sparta et al., 2012). Mice were allowed to recover for 2–3 weeks before testing began.

Photo-stimulation was always bilateral. A custom-made commutator was used to split a single laser beam into two beams for bilateral stimulation. During stimulation sessions, mice were connected to a 473-nm wavelength laser by two sheathed fibers (62-μm core diameter, connected by ceramic sleeves, Precision Fiber Products). The total output of the laser was adjusted each day, to obtain ~636 mW/mm^2^ transmittance.

Following completion of experiments, mice were anesthetized with isoflurane and perfused with ice-cold 4% paraformaldehyde. Brains were post fixed for ~24 h at 4°C, cryoprotected in sucrose solution, and then sliced at 60 μm on a Vibratome. Slices were incubated with primary chicken anti-GFP (1:1000, AbCam) and TH primary rabbit anti-TH (1:1000, Millipore) with 10% goat serum and 0.25% Triton X-100 overnight at 4°C. Secondary antibodies (Alexa Fluor 594 goat anti-rabbit and Alexa Fluor 488 Goat anti-Chicken) were used to visualize TH and GFP, respectively (1:250, Molecular Probes). Slices were imaged with an Axio Zoom.V16 (Zeiss) microscope and processed using Zen software (Zeiss).

## Results

### Video tracking

The sucrose spout was located next to the platform on which the mouse stands. Following the cue, the mouse started a movement toward the sucrose spout, adjusting its body to prepare for the reward delivery. Once the sucrose solution was delivered, the mouse made another movement to consume the sucrose. Because the mice were not restrained and allowed to move freely within the confines of the small platform, the movement trajectories varied considerably between animals. Although we used a single LED placed on the head to track movements, this does not mean that the mouse only moved its head. There was clear movement of the whole body, as confirmed by pressure pads placed underneath the mouse, and video tracking of tail movements at the time of cue or reward (Figure 1E).

### Classification of DA neurons

**Figure 2:**
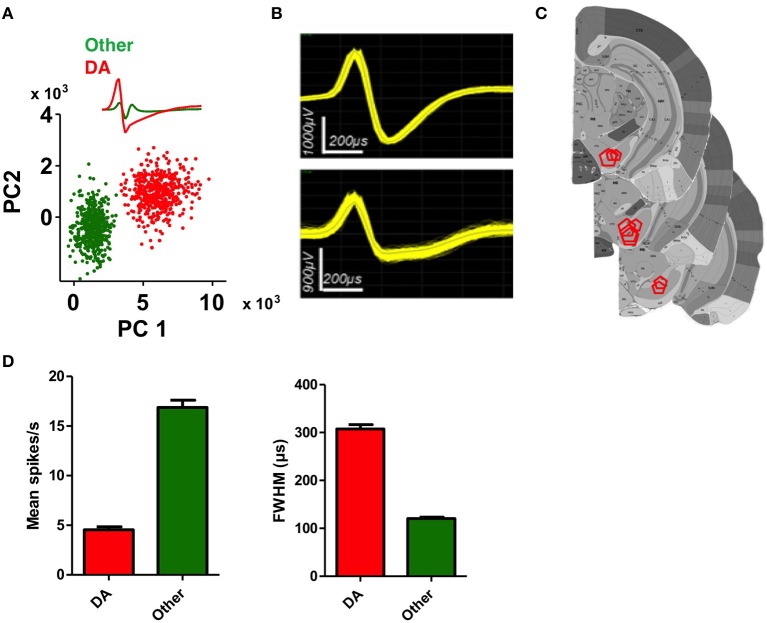
**Identification of DA neurons in the substantia nigra. (A)** Classification of a putative DA neuron and a non-DA neuron using principal component analysis (PCA). **(B)** Representative waveforms of putative DA neurons. **(C)** Summary of electrode placements shown in coronal brain sections take from the Allen Brain Atlas (Lein et al., 2006). **(D)** Average firing rate and spike width (FWHM, full width at half maximum) of putative DA neurons and non-DA neurons. DA neurons are characterized by lower firing rates and wider spike widths (unpaired *t*-tests, *ps* < 0.0001).

We recorded activity of DA neurons in the SNc, the largest DA cell group targeted by classic studies of phasic DA activity (Ljungberg et al., 1992; Schultz, 1998a). As shown in Figure 2, putative DA neurons recorded from the SNc, are identified by their firing rate and waveforms (Grace and Bunney, 1984; Rossi et al., 2013a,b). DA neurons have considerably wider spike waveforms and overall lower firing rates compared to other neurons in this area (Figure 2D, unpaired *t*-tests, *ps* < 0.0001). We recorded from 106 putative DA neurons over a period of 1–6 months depending on the animal. The rest of the recorded waveforms are often putative GABAergic neurons due to their narrow spikes and high firing rates.

### Correlation between DA activity and kinematics

**Figure 3:**
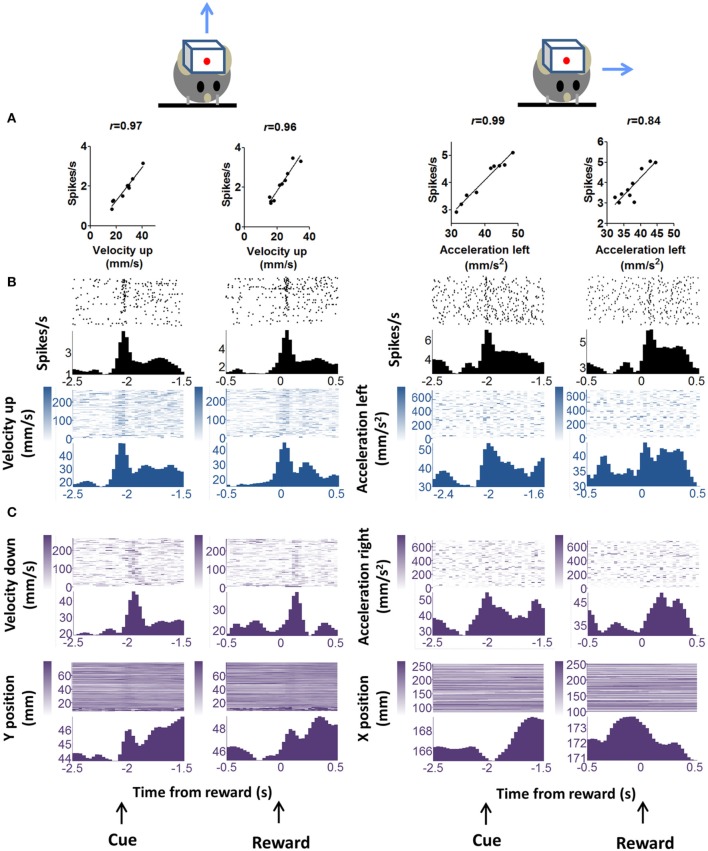
**“Burst” DA neurons show positive correlation with kinematic variables. (A)** Firing rate of representative neuron showing positive correlation with vector components of velocity and acceleration. Two major movements are detected during the trial, one in response to the cue and the other in response to reward delivery. These are displayed separately. “Velocity up” means velocity in the upward direction. Blue arrows indicate movement direction, but note that only the vector components are indicated. Actual movements would consist of both x and y components. The correlation analysis uses data displayed in the raster plots below. A 1 s peri-event window (either cue or reward) was used. **(B)** Peri-event raster plots of the neurons and the correlated kinematic variables. **(C)** The major alternative kinematic variables are shown. These are not highly correlated with neural activity as determined by our unbiased cross-correlation analysis.

**Figure 4:**
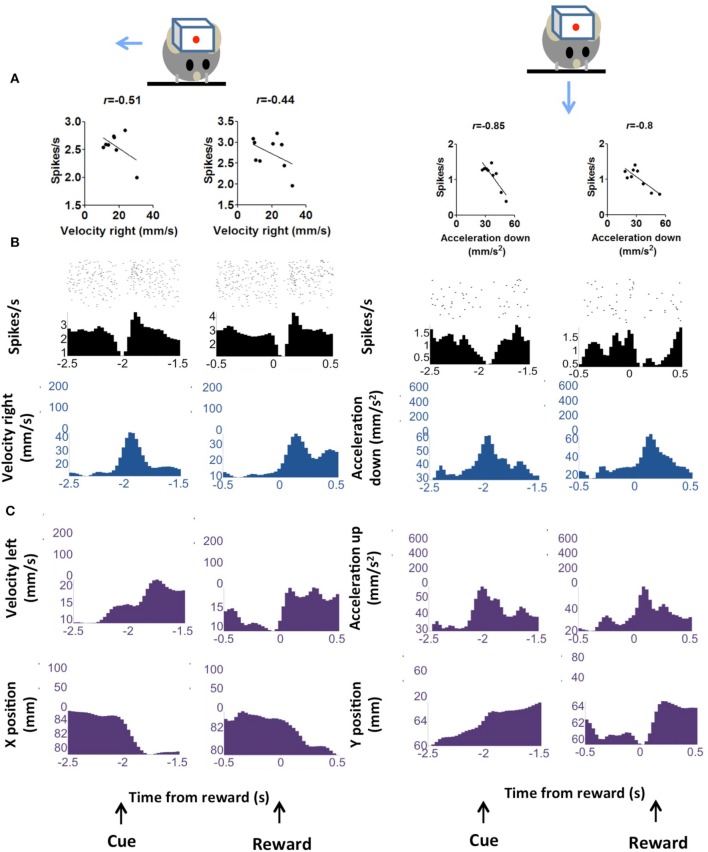
**“Pause” DA neurons is negative correlated with kinematic variables. (A)** Firing rate of representative neuron showing negative correlation with vector components of velocity and acceleration. Two major movements are detected during the trial, one in response to the cue and the other in response to reward delivery. These are displayed separately. **(B)** Peri-event raster plots of the neurons and the correlated kinematic variables. **(C)** The major alternative kinematic variables are shown.

We observed phasic activity of DA neurons following the auditory cue and sucrose reward delivery (Figure 3), similar to what was observed in previous studies in monkeys (Schultz et al., 1997) and in mice (Cohen et al., 2012). But we found a strong correlation between the neural activity and kinematics at the time of the auditory cue or the reward. The correlation analysis shown in Figures 3, 4 was performed on neural and movement data taken from a 1 s peri-event window for the task related events—either cue or reward. However, the same conclusion was confirmed by an unbiased analysis using data from the entire session. It is important to note that our classification of different functional classes of neurons is based on the unbiased cross correlation analysis from the entire session.

**Figure 5:**
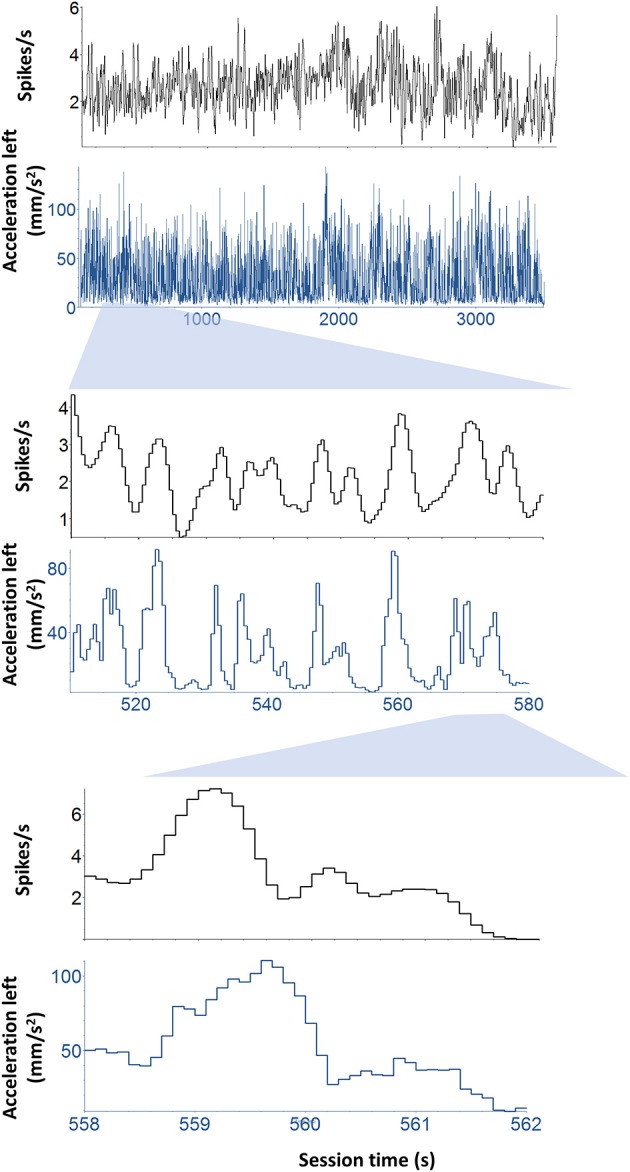
**Continuous correlation between neural activity and kinematics**. Illustration of a representative neuron and its correlation with kinematics independent of task-related events such as cue and reward. To dissociate kinematic variables from these task events. Rather than selecting only data from the trial, we performed an unbiased correlation between firing rate and the kinematic variables for the entire session, including inter-trial-intervals. This unbiased analysis was used to classify the neurons.

The firing rates of DA neurons at the time of cue and reward were correlated with movement velocity and acceleration. Because the movements were time-locked to the cue and reward delivery, it is important to dissociate kinematic variables from these task events. For this reason, rather than selecting only data from the trial, we performed an unbiased correlation between firing rate and the kinematic variables for the entire session, including inter-trial-intervals (Figure 5). The strength of overall correlation cross correlation does not depend on the high correlation during the trials. By far the majority of the data come from inter-trial-intervals (20–50 s) because the latter last much longer than the trial duration (~2 s). The correlations between DA activity and kinematics are significant after controlling for the effects of event timing and reward related responses.

This unbiased method, using cross-correlation of data from the entire session, was used to classify the neurons (Table 1). Note that, because different vector components of velocity and acceleration variables can be correlated, there are often statistically significant correlations between DA activity and multiple vector components, but the kinematic variable with the highest correlation coefficient was used to classify the neurons. This classification method supports the observation of strong correlation between DA firing and movement at the time of cue and reward. Therefore, a given neuron shows similar correlation during the trial (following cue or reward) and during the inter-trial-interval, when spontaneous movements were observed.

**Table 1:** **Summary of different types of DA neurons**.

	**Velocity**	**Acceleration**	**Total**
Up	18	9	31
Down	19	13	28
Left	13	4	18
Right	16	5	20
Total	66	31	97

**Figure 6:**
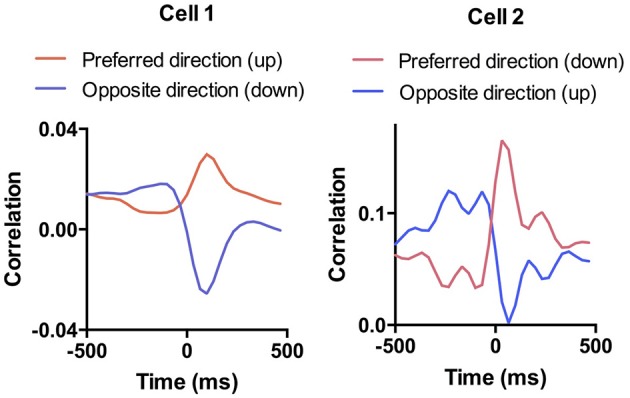
**Selectivity of DA responses**. To illustrate the direction selectivity of DA neurons, we compared the session-wide cross-correlation between neural activity and velocity in opposite directions. Shown are two examples in which the cell is positive correlated with movement in one direction and negatively correlated with movement in the opposite direction. This pattern is similar to what we observed previously in SNr GABAergic output neurons (Barter et al., 2015).

Cross-correlation analysis allows us to examine the direction specificity of the relationship between neural activity and movements. If a neuron is positively correlated with velocity in a particular direction, it will often show the opposite correlation with velocity in the opposite direction (Figure 6). Thus, not only do DA neurons show direction selectivity, there also appears to be a reciprocal inhibition organization, with opponent signals generated for different movement directions. This pattern was not common in neurons that are negatively correlated with movement in a particular direction.

### Appetitive vs. aversive behavioral tasks

**Figure 7:**
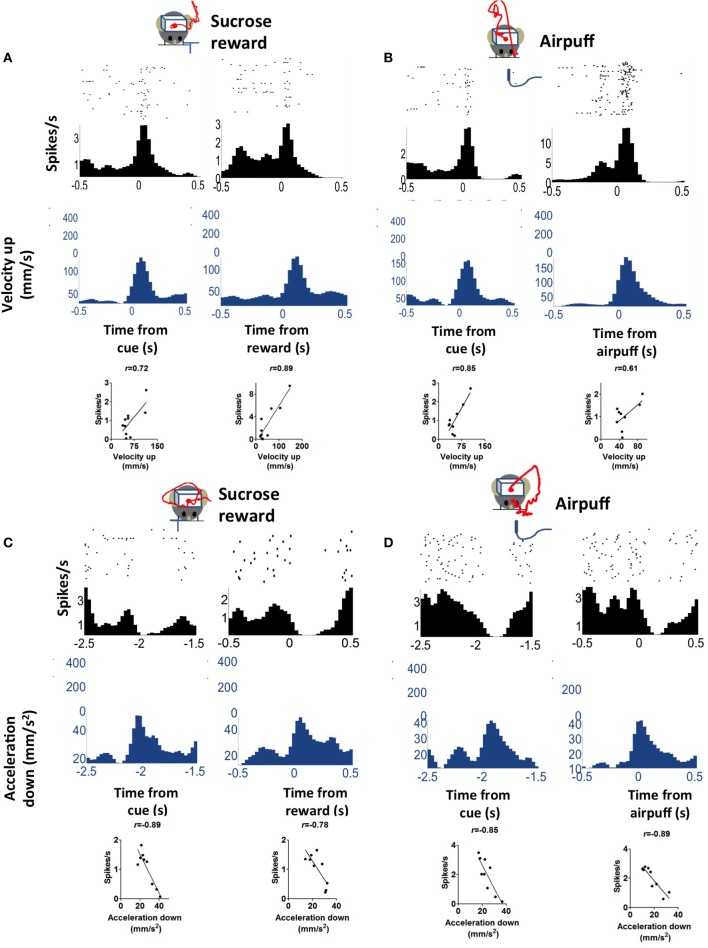
**Correlation between firing rate and acceleration is similar on appetitive (sucrose reward) and aversive (air puff) trials**. **(A)** An example of a positively correlated DA neuron on reward trials. The red line represents average movement trajectory from the session. **(B)** The same neuron on air puff trials. Note that the actual trajectories differed significantly between reward and air puff trials, but the upward components of velocity are similar, as shown here. **(C)** An example of a negatively correlated DA neuron on reward trials. **(D)** The same neuron on air puff trials.

Because we used an appetitive behavioral task, it is unclear whether the observed correlation between neural activity and acceleration is specific to reward-related behaviors, whether in reward anticipation or consumption. To address this question, we performed additional experiments with aversive outcomes. We measured the activity of DA neurons on both appetitive and aversive conditioning trials. The same experimental setup was used, with the same trace conditioning procedure, except an aversive air puff was delivered as the unconditional stimulus instead of sucrose solution, from the same location where sucrose was delivered (*n* = 3 mice). On these aversive trials, the behavior was very different from that observed on sucrose reward trials, as clearly revealed in the movement trajectories. The animal moved away from the site of the air puff. Following the cue, the animal attempts to avoid the expected air puff by moving its body, and again at the time of the air puff delivery. On such trials, the correlation between kinematics and neural activity was still robust, and comparable to the correlation on reward trials (Figure 7).

### Summary of correlation analysis

Based on data from all recording sessions (appetitive as well as appetitive/aversive), we identified neurons that are correlated with velocity and acceleration in four directions (Table 1). Neurons associated with different directions have comparable firing rates [One-Way ANOVA, *F*_(3, 97)_ = 0.63, *p* = 0.6]. Of all recorded neurons, nine neurons were not significantly correlated with any of the kinematic variables. The firing rates of most recorded DA neurons (97/106, 91%) were correlated with either movement acceleration or velocity. Both positive and negative correlations were found, but the positive correlation is far more common (78 vs. 22% of correlated neurons).

**Figure 8:**
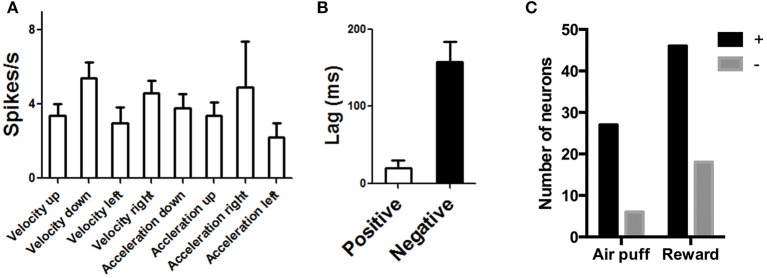
**Population data for DA neurons on rewarded and air puff trials. (A)** Different classes of DA neurons show comparable firing rates. **(B)** Using cross correlation analysis, we also found the lag is much longer for negatively correlated neurons, suggesting that, in these neurons, a pause in firing precedes some movement.**(C)** The proportion of positively and negatively correlated neurons is similar for aversive and rewarding sessions.

The DA neurons with positive correlation with velocity or acceleration (Figure 3) are the burst neurons that increase firing transiently at the time of salient events (cue or reward), which have been well-documented in previous studies (Schultz, 1998a). The less common neurons with negative correlations are those that pause at the time of cue or reward (Table 1). The different types of neurons all show comparable firing rates [Figure 8A, One-Way ANOVA, *F*_(7, 89)_ = 1.30, *p* = 0.26]. The lag between neural activity and kinematics is much longer in negatively correlated neurons (Figure 8, 158 ± 25.7 ms, compared with 20.0 ± 10.5 ms for positively correlated neurons, unpaired test, *p* < 0.0001). The proportion of positively correlated and negatively correlated neurons is not different for appetitive and aversive sessions (Figure 8C; *chi-square* = 1.16, *p* = 0.28).

### Lateralization of the DA response

**Figure 9:**
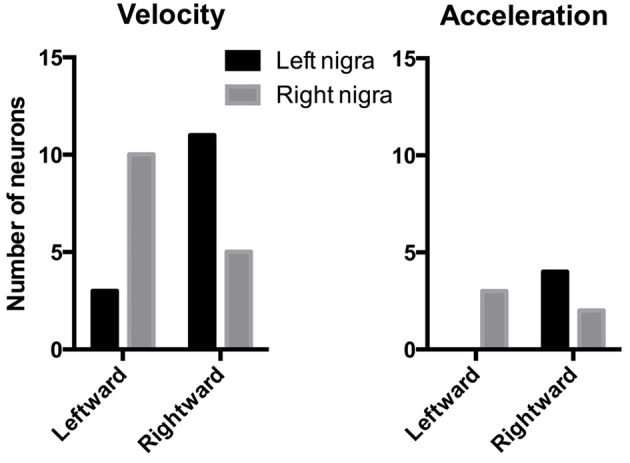
**Lateralization of direction-specific neurons**. Among velocity-related DA neurons, there are more rightward neurons in the left nigra, and more leftward neurons in the right nigra. There was no significant lateralization among acceleration-related neurons, though the sample size is much smaller.

The electrode arrays were always implanted unilaterally, in the left nigra in six mice, and in the right nigra in five mice. Of the movement-correlated neurons, 40 were recorded from the left nigra and 57 from the right nigra. Since many neurons recorded were correlated with either leftward or rightward movements, we examined the distribution of these neurons to see if there is any lateralization of leftward and rightward neurons. As shown in Figure 9, among velocity-correlated neurons, most DA neurons correlated with rightward movements are found in the left nigra, whereas most DA neurons correlated with leftward movements are found in the right nigra (*Chi-square* = 5.99, *p* = 0.01). There was no statistically significant difference between the two sides for acceleration-correlated neurons (*Chi-square* = 3.60, *p* = 0.06), though the sample size is much smaller compared to the velocity neurons.

### Optogenetic stimulation of DA neurons

**Figure 10:**
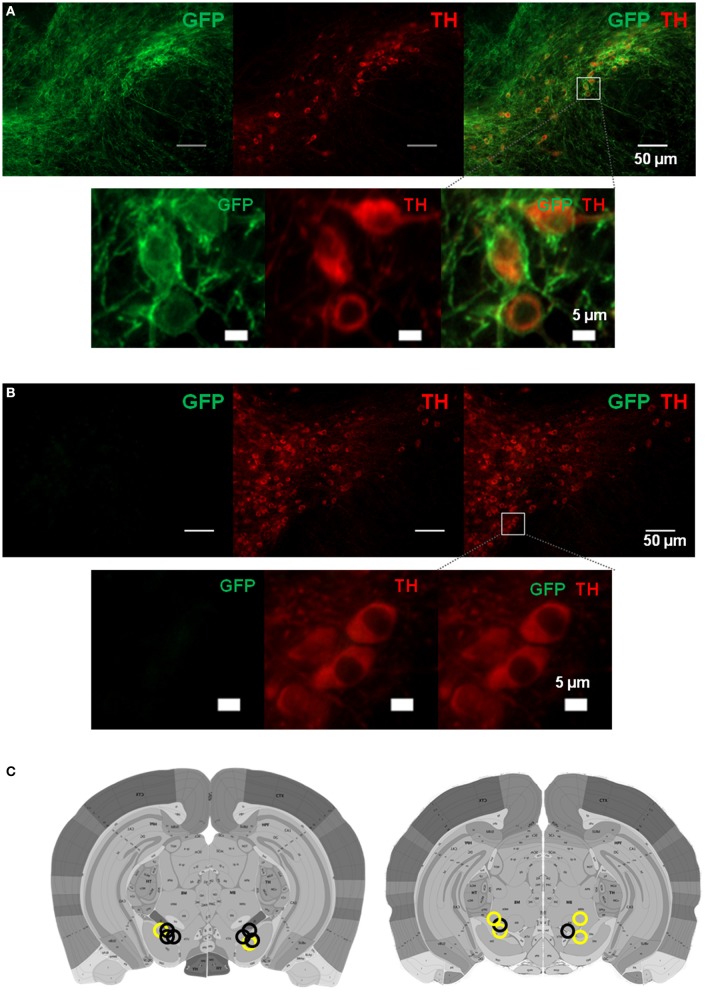
**Expression of channelrhodopsin 2 in dopamine neurons in** ***Th::Ai32*** **mice. (A)** Locations of bilateral optic fibers based on histological verification of coronal brain slices. Representative GFP fluorescence, indicating ChR2 expression, is colocalized with TH in the substantia nigra of *Th::Ai32* transgenic mice. Scale bar is 50 μm (upper panels). Lower panels are zoomed in images from the box shown in the upper right panel (scale bar 5 μm). **(B)** GFP fluorescence is absent in *Th-Cre* control mice. Same conventions as (**A**). **(C)** Optic fiber placements for *Th::Ai32* (*n* = 4; black circles) or *Th-Cre* (*n* = 3; yellow circles) mice. Atlas images are from the Allen Brain Atlas (Lein et al., 2006). Available from: http://mouse.brain-map.org/.

Although our electrophysiological experiments show striking correlations between movement kinematics and firing rates of DA neurons, such results do not tell us whether DA activity is directly involved in generating movement. To establish a “causal” role for DA neurons in movement, it would be necessary to manipulate the activity of these neurons while measuring movement kinematics. To selectively stimulate DA neurons, we developed a transgenic mouse line in which channelrhodopsin 2, which depolarizes neurons upon stimulation with blue light (Boyden et al., 2005), is expressed selectively in DA neurons. This was accomplished by crossing *Th-cre* (expressing Cre recombinase in tyrosine hydroxylase positive neurons) with the *Ai32* line, which has a floxed stop cassette at the *Rosa26* locus, allowing Cre-inducible ChR2 expression (Madisen et al., 2012). Representative pattern of ChR2 expression is shown in Figure 10.

We used a blue laser (473 nm) to stimulate two groups of mice, one group with ChR2 expressed in Th-positive neurons (*Th::Ai32*), and a control group with only Th-Cre expression but no ChR2. We found that optogenetic stimulation of DA neurons could induce movements. Although such movements were sometimes too subtle to be apparent to a casual observer, they could easily be detected by our tracking program. Because the different types of DA neurons are functionally defined, it is not yet possible to manipulate each type independently and examine the impact on movement kinematics. As photo-stimulation presumably activated multiple types of DA neurons simultaneously, the net effect will not be pure vertical or horizontal movements. We therefore used distance and speed to quantify the movements. Distance is defined as the distance traveled between the location of the LED at the onset of stimulation train and its location at the termination of the train. Speed is defined as the derivative of the distance.

**Figure 11:**
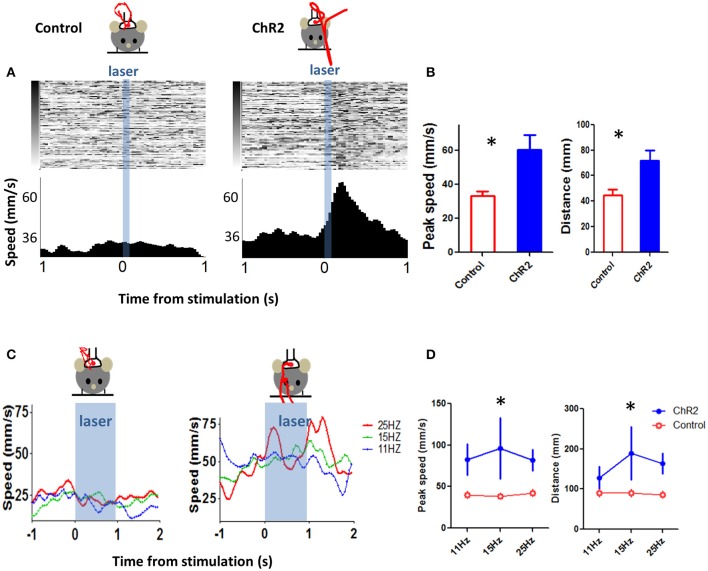
**Optogenetic stimulation of DA neurons can elicit movements**. **(A)** To mimic burst firing of DA neurons, we selectively stimulated DA neurons using optogenetics. We generated a transgenic mouse line (Th-Cre × Ai32) to selectively express ChR2 in DA (tyrosine hydroxylase-positive) neurons. A brief stimulation at 40 Hz (3 ms pulse width, 5 pulses) generated movement, in the ChR2 (Th::Ai32) mouse but not in a control mouse (Th-Cre) that also received the same light stimulation. Control mice were implanted with fibers and stimulated using identical procedures. Red trace represents movement trajectory. **(B)** Peak speed and distance for ChR2 (Th::Ai32) and control (Th-Cre) mice. ^*^p < 0.05. **(C)** Left, movement kinematics plotted for different stimulation frequencies (11, 15, and 25 Hz, 3 ms pulse width, 1 s duration. **(D)** Peak speed during stimulation train and distance traveled (at the end of the train) at different stimulation frequencies. ^*^p < 0.05.

As shown in Figure 11, we first mimicked the phasic burst of activity by high frequency stimulation (40 Hz, 5 pulses, 3 ms pulse width, power ~636 mW/mm^2^). This stimulation parameter generated movements that are similar to what we observed during our recording experiments (2 *Th-Cre* control mice, eight sessions, 3 *Th::Ai32* mice, 14 sessions, 40 stimulation trials per session, variable inter-trial-interval: 6–18 s with a mean of 12 s, unpaired *t*-test, *p* = 0.025 for peak speed, and *p* = 0.028 for distance).

**Figure 12:**
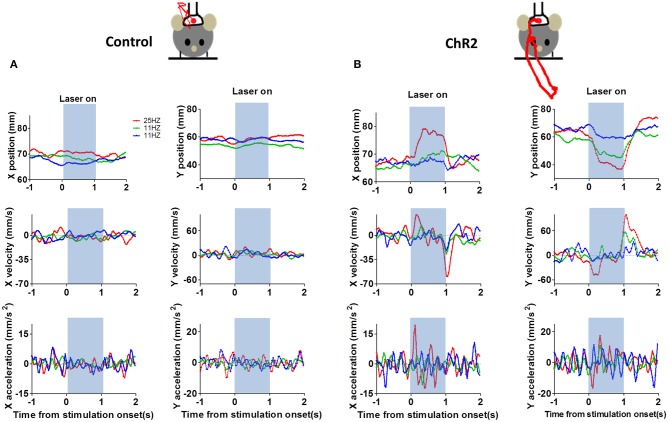
**Detailed movement kinematics during optogenetic stimulation**. **(A)** Representative horizontal (x) and vertical (y) components of the movements in a control mouse (*Th-Cre*). Position, velocity, and acceleration are plotted separately. Red traces show movement trajectories produced by the stimulation. **(B)** Representative data from a *Th::Ai32* mouse.

We then varied stimulation frequency (each train lasted ~1 s) and examined the effects on movement kinematics (3 *Th-Cre* control mice, 12 sessions, 4 *Th:: Ai32* mice, nine sessions, 8–20 stimulation trials per session, inter-trial-interval = 9 s). To analyze the movement kinematics generated by photo-stimulation of DA neurons, we used a repeated-measures Two-Way ANOVA with genotype and stimulation frequency as factors. For peak speed during stimulation, we found a main effect of genotype [*F*_(1, 38)_ = 6.44, *p* = 0.02], no main effect of frequency [*F*_(2, 38)_ = 0.2, *p* = 0.82], and no interaction between genotype and frequency [*F*_(2, 38)_ = 0.41, *p* = 0.67]. For distance moved, we found a main effect of genotype [*F*_(1, 38)_ = 6.06, *p* = 0.02], no main effect of frequency [*F*_(2, 38)_ = 0.90, *p* = 0.41], and no interaction between genotype and frequency [*F*_(2, 38)_ = 0.92, *p* = 0.41]. These results demonstrate that stimulation of DA neurons is sufficient to generate movements. The distinct horizontal and vertical components of the kinematic variables produced by stimulation are shown in Figure 12. This example showed that movements induced by photo-stimulation had both x and y components, in accord with our prediction that stimulation will affect multiple types of DA neurons.

### Comparing DA and GABA neurons

**Figure 13:**
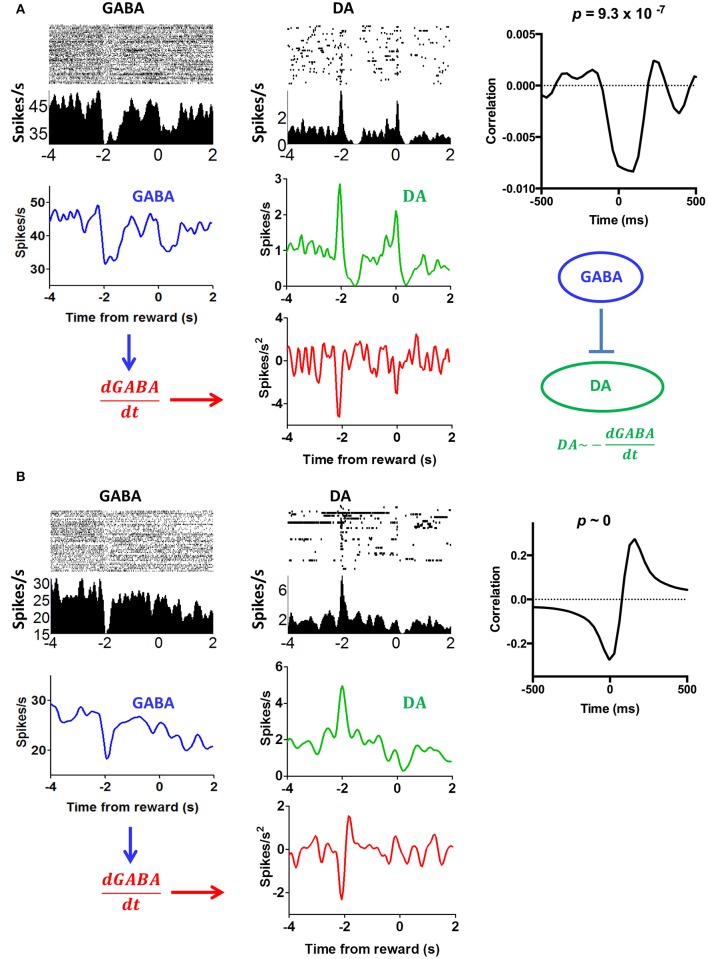
**Comparison of putative nigral GABA and DA neurons from the same electrode array. (A)** The activity of the GABA neuron reflects y position coordinates, an example of representation of instantaneous position coordinates reported in our recent study (Barter et al., 2015). The activity of DA neuron reflects velocity in the upward direction. If we take the derivative of the GABA output, we can generate a mirror image of the DA activity. This result, then, is in support of disinhibition: the reduction in GABA output is accompanied by an increase in DA firing. Note that these projections are mostly collaterals of fibers terminating in other areas such as the tectum and thalamus. Cross-correlogram shows the relationship between DA firing and the derivative of the GABA output from the entire session. **(B)** Another example illustrating the relationship between DA and GABA neurons from a different mouse.

SNr GABA output neurons are known to inhibiti DA neurons. Some have argued that the firing of DA neurons is largely due to disinhibition: bursting is observed when GABA output neurons pause (Tepper and Lee, 2007; Paladini and Roeper, 2014). This view is supported by our data. In some sessions, we simultaneously recorded from putative DA neurons neurons and GABA neurons from the same electrode array. Of these putative GABA neurons, 20 are also correlated with head position coordinates (x or y), as we previously described (Barter et al., 2015). Figure 13 shows examples of simultaneously recorded DA and GABA neurons, which are both correlated with movement along a single axis (y axis). When a DA neuron is compared with a neighboring GABA output neuron, there is a clear mathematical relationship between their outputs, as shown by the cross correlation analysis. It appears that the derivative of the GABA output signal is subtracted from the DA output.

## Discussion

Using a Pavlovian trace conditioning task, we first studied the responses of DA neurons following cue and reward presentation. Such responses are similar to what was reported in previous work on monkeys (Schultz, 1998b), rats (Roesch et al., 2007), and mice (Cohen et al., 2012). At first glance, our results may appear to support the idea that phasic DA neurons encode reward prediction errors (Schultz et al., 1997), but such an appearance is misleading, for a careful analysis revealed for the first time a continuous relationship between the firing rates of DA neurons and specific kinematic variables.

A key difference between our study and previous work is our use of continuous video tracking to quantify the behavior of the animal, allowing us to examine the relationship between single unit activity and movement kinematics. As shown in Figure 1, the mouse typically initiates a movement following the cue, and again following the reward delivery. The single unit activity is highly correlated with the vector components of the kinematic variables (velocity and acceleration). Even though most DA neurons showed phasic activity at the time of cue presentation or reward delivery, this pattern can be explained by the actual movement kinematics of the animal. The neural representation of kinematics is found not only at the time of cue or reward, but also during the inter-trial-interval (Figure 6). Different types of DA neurons are identified based on their correlation with movement kinematics during the entire behavioral session. As shown in Figure 7, the correlation is also independent of outcome valence–whether it is aversive (air puff trials) or rewarding (sucrose trials); a neuron that fires during upward movement on a sucrose trials also shows the same correlation on an air puff trial. DA neurons thus represent movement kinematics whether the movement was performed to acquire sucrose or to avoid air puff.

To ascertain the “causal” role of DA neurons in movement, we also mimicked phasic DA activity by photo-stimulation. Using brief stimulation pulses and physiological frequencies (Pan et al., 2013; Rossi et al., 2013b), we were able to elicit movements that are comparable to those during our recording experiments (Figure 10).

### Caveats

Our results therefore suggest that DA is critical for shaping the kinematics of movements, and support the hypothesis that nigrostriatal DA modulates the gain of a closed loop movement velocity controller (Yin, 2014b). They have important implications for our understanding of BG function, but before discussing these implications, a few caveats must be mentioned at the outset.

First, we only examined the activity of nigrostriatal DA neurons in the SNc, which mainly target the dorsal striatum. We did not record from the mesolimbic DA neurons in the ventral tegmental area (VTA), which target the ventral striatum, prefrontal cortex, and other limbic regions. It is possible that VTA DA neurons have different properties, which allow them to encode reward prediction errors. Although we cannot rule out this possibility, classic studies from monkeys concluded that DA neurons from both SNc and VTA have similar phasic responses in relation to behavioral events (Schultz, 1998a). In addition, recent studies also found a strong correlation between VTA activity and movement (Puryear et al., 2010; Wang and Tsien, 2011b; Wang et al., 2013). It remains to be seen whether VTA DA neurons also represent movement kinematics.

In our optogenetic experiments, the Cre driver line which we used for generating the *Th::Ai32* mice may express Cre recombinase in some non-DA neurons as well (Lammel et al., 2015). Although the main evidence showing Cre expression in non-DA neurons comes from the VTA rather than the SNc, we cannot rule out the possibility that photo-stimulation activated a few non-DA neurons as well. Although our histological analysis showed that ChR2 expression is confined to tyrosine hydroxylase positive neurons, exhaustive cell counts using confocal images will be necessary to confirm this conclusion.

Pan and colleagues recently reported a subset of optogenetically identified SNr GABAergic neurons with short latency burst responses to salient cues during a trace conditioning task (Pan et al., 2013). Are the putative DA neurons reported here just a subset of GABA neurons with phasic responses? In the absence of cell type identification in each recorded neuron, we cannot rule out this possibility. But it is unlikely for a number of reasons. First, the average firing rates of putative DA neurons (usually ~5 Hz) reported here are comparable to those of identified DA neurons reported in Pan et al. The high tonic firing rates of their GABA neurons are also comparable to what we observed, indicating similar cell type classification criteria. Indeed, in recent studies we also observed the type of short latency phasic responses in SNr GABA neurons, identified using similar criteria as the present study (Fan et al., 2012; Rossi et al., 2013a). In addition, Pan et al. did not examine the relationship between neural activity and movement kinematics, leaving open the possibility that their observation of reduced firing rates of DA neurons following extinction can be explained by the reduction in Pavlovian conditional responses (licking in their case) or other more specific changes in unobserved movement kinematics.

### Reward prediction error

Previous work found burst firing of DA neurons following reward delivery, but this phasic activity occurred earlier in time with training, following any reward predicting cue (Schultz, 1998a). This observation was thought to support models of learning that use a prediction error as a teaching signal (Schultz, 1998b). It is beyond the scope of this article to discuss problems with learning models that use prediction errors, but our results clearly furnish evidence against the reward prediction error hypothesis of DA function.

Previous studies in support of this hypothesis rarely quantified movements, even though reward-related behavioral variables are not dissociated from movement kinematics. Moreover, most studies used restrained animals; even if they attempted to move by generating the requisite neural signals, it would not have been possible to achieve the actual movements. Inability to move does not mean that the neural signals necessary for movement velocity control are absent. Consequently, in all previous studies on reward prediction errors, there is an important movement confound.

Our results suggest that the previously observed shifts in phasic DA activity over time could reflect changes in movement kinematics. Of course, without using the identical experimental design, a direct comparison between our results and those from previous studies is impossible. Yet none of the standard manipulations (e.g., of reward size, probability, violations of reward prediction) used in previous studies can be free of the movement confound. In all these cases, the experimental manipulations can produce behavioral differences too subtle to be noticed by the casual observer. But we do not know how the animal is moving under the different experimental manipulations.

The reward prediction error hypothesis cannot explain the selectivity of DA neurons for different directions of motion (Figure 3), the linear relationship between DA activity and kinematic variables independent of reward (Figure 6), the high correlation between DA activity and movement on appetitive and aversive trials (Figure 7), the lateralization of leftward and rightward selective neurons on the two sides of the brain (Figure 9), and the observation that DA activity reflects the mathematical derivative of the GABAergic SNr output (Figure 13). Nor can it explain previous observations on locomotion (Wang et al., 2013), posture control (Barter et al., 2014), motivational modulation (Rossi et al., 2013a), or instrumental actions (Jin and Costa, 2010; Fan et al., 2012). Yet, as we shall see below, all these results can be explained by the crucial role of DA in shaping movement kinematics.

### Nigrostriatal DA and control of movement velocity

The correlation with vector components of velocity and acceleration observed in DA neurons is similar to what we observed in striatal neurons (Kim et al., 2014). This is not surprising since the striatum is the major target of DA projection, and stimulation of the DA neurons or of their targets in the striatum can elicit movements (Ferrier, 1876; Kravitz et al., 2010; Rossi et al., 2015).

Our finding of direction specificity in DA neurons and striatal neurons suggests considerable specificity in the pattern of nigrostriatal projections. DA neurons that fire during leftward movements, for example, are hypothesized to project to striatal neurons that fire during leftward movements. Unilateral stimulation of the striatum or application of DA agonists after depletion-induced receptor supersensitivity can produce contraversive turning or circling behavior (Ferrier, 1876; Hefti et al., 1980; Rossi et al., 2015). The striatal modules and their corresponding DA neurons are expected to be asymmetrically distributed across the two sides, e.g., the right striatum and nigra are expected to contain more units that generate leftward movements. This prediction is supported by our results, which show that the left nigra contains more rightward neurons and the right nigra contains more leftward neurons (Figure 9).

Any point in space can be described in Cartesian coordinates, which indicate the direction and distance of this point from some arbitrarily chosen origin. In our model, the origin is the current position of the animal. From the egocentric reference frame, the position change is generated by the action of different classes of striatal neurons, but through a velocity control mechanism.

Movement direction is often assumed to be encoded by a population vector, which combines activity from many motor cortical neurons (Georgopoulos et al., 1986). On the other hand, here we assume that such tuning properties reflect the presence of different classes of neurons for different vector components. Their outputs can be combined by vector addition. Neurons that fire preferentially for a specific direction command a downstream controller that will ultimately produce movement in this direction. For movement along any axis (x, y, z), opponent signals are needed for antagonistic controllers for opposite directions. This is supported by previous work suggesting distinct controllers in the brainstem for horizontal and vertical movements in orienting movements (Masino, 1992).

### Transition control

The role of DA in scaling performance has long been noted and emphasized by another class of ideas, e.g., DA is thought to reduce sensorimotor threshold (White, 1986), or increase “response vigor” (Niv et al., 2007), or bias action selection (McClure et al., 2003; Roesch et al., 2007). Such ideas just re-describe the behavioral observations following DA manipulations. They do not offer any mechanisms by which DA can affect BG circuit function.

On the other hand, the present results support the model that the BG circuit acts as a transition controller, with DA acting as the gain of this system. Transition, in the sense used here, refers to *change* in specific perceptual representations, which range from modality specific perceptual representations to multimodal representations of more abstract relationships. To control these perceptions, a hierarchy of negative feedback control system is used, in which variable outputs are produced to reach and maintain desired inputs, as discussed below.

A major function of the sensorimotor BG circuit is to control transition in body configurations, which corresponds to movement velocity. This circuit controls the rate of change in proprioceptive transitions by sending descending reference signals to position controllers in the midbrain and diencephalon, which in turn command muscle length and joint angle controllers in the reticulospinal pathway (Yin, 2014a).

The striatum contains comparators that compare reference and input velocity values, and the resulting velocity error signals are integrated and converted into the nigral output, which represents the reference signals for lower level position controllers.

### Position control

It is critical to view the BG output as a continuously varying quantity, rather than either on or off. At any time, the output from SNr GABA neurons represents a specific body configuration and orientation. Constant firing rate is associated with a constant reference signal, i.e., no change in posture. When the firing rate does not change, the posture is fixed. To generate a voluntary action, the reference signal can be adjusted by a change in BG output from the SNr GABA neurons. For example, one change associated with steering of the body is a gaze shift, as the foveation target shifts to a different position. The descending command allows steering in any direction by shifting to a new target position. This allows acquisition of any sensory input with the corresponding changes in body configurations.

There are multiple position controllers, found in the tectum, pedunculopontine nucleus, and perhaps the thalamus, that receive descending commands from the BG output. Without a change in the descending BG output, lower level position controllers can independently control position, based on their own reference signals. They can produce outputs to resist any perturbation, even when there is no overt movement. Their outputs vary continuously according to the perturbation signals transmitted via the feedback path. A large output is due to a significant perturbation, as the input transiently deviates from that allowed by the internal reference. This is usually created by a salient stimulus (visual, auditory, or somatosensory), and the resulting behavioral response is traditionally labeled “reflexive.” So-called reflexes are behavioral outputs generated to restore the desired inputs dictated by lower level reference signals, for position, muscle length, tension, etc. Interestingly, artificially induced position error signals from deep layer tectal neurons can also activate DA neurons (Dommett et al., 2005). This type of short latency activation may serve to prime the higher levels for transition control.

One important property of the different types of position controllers is that they will generate movement at a certain velocity, proportional to the position error. For a given position error, the velocity will not vary much. In other words, for behaviors generated at levels below the BG, velocity cannot be regulated independently. To change the velocity, one must change the rate of change in their reference signals, which dictates how quickly the position input should change. This requires a higher level system, a master loop in a cascade control hierarchy. It can be achieved by placing the velocity controller above the position controller, so that the output of the higher loop is the reference signal for the lower loop (**Figure 15**). In the output function of the velocity controller, there is a neural integrator that converts the striatal output signal into the rate of change in the nigral output. Consequently, the rate of striatal neurons reflects velocity, yet their target nigral GABA neurons show position-related activity. Once a new position is achieved, it is held in the absence of further input from the striatal neurons.

### Commanding the velocity controller

Ascending the hierarchy still further, we must ask what is represented by inputs to the striatum. According to our model, corticostriatal inputs reaching the sensorimotor comparator function in the striatum can either be perceptual representations or reference signals (**Figure 15**). This arrangement allows the velocity controller to be commanded by any number of systems, just as a particular action can be performed for any number of purposes, e.g., turning left to reach a reward or to avoid an air puff (Kim et al., 2014). The velocity controller can therefore be viewed as the final common path for behaviors that are traditionally called “voluntary.” These behaviors originate from error signals in controllers above the level of velocity control. The higher error signals can become the reference signals for the velocity controller. The magnitude of the signal will be reflected in the actual movement velocity, unless two conflicting commands are sent simultaneously.

Many reference signals at the higher levels are acquired perceptual representations, i.e., memories. These signals represent behavioral goals, yet goals, in the sense used here, do not represent nor determine desired outputs. Instead they represent perceptual variables to be achieved.

### Control of input

To understand the hierarchical control model, it is important to grasp the fundamental difference between this model and traditional models that purport to use closed loop control (Todorov and Jordan, 2002). Attempts to apply control theory in neuroscience have failed so far, largely due to a key misunderstanding introduced by engineering terminology. In the engineering tradition, controllers are typically said to control their outputs, because the output value can match the desired value (from the perspective of the engineer), which is fed into the system. The controller is treated just like an input/output device: reference signal is injected and the desired output is produced.

Thus, according to the traditional view, feedback control means computing the commands (or “control signals”) needed to produce a particular position and then executing these commands. But this analysis is very misleading. Engineers measure the output of a negative feedback controller and compare the measurement with the desired reference value to generate the error signal needed to produce the output. The desired output is in fact some measure of the actual output. Such systems are known as servos, for without their own reference values they can never be autonomous agents. But the prescribed reference value in any living organism does not come from without, say a user adjusting the temperature setting or commanding a position, but from within (Powers et al., 1960). The measure of its own output is also taken by the organism itself, via continuous feedback detected by sensory systems. Consequently, in living control systems, the reference signal is never a input command from the environment. The true input always comes from perceptual representations, the values of which, at any moment, are dictated by the intrinsic reference signals within the organism. The key operation, then, is the control of inputs, while varying behavioral outputs. The variable outputs are only the outward manifestation of the more fundamental process of control, which remains largely invisible to the casual observer. Whenever feedback control is viewed as the control of output, the control loop cannot be analyzed correctly, because the inside and outside of the system are reversed. This misunderstanding led to widespread misunderstanding of gain and feedback delays in closed loop controllers, as well as conclusions that the nervous system contains internal models of Newtonian laws, when what is measured is just environmental properties fed back to the organism (Robinson, 1981; Green and Angelaki, 2010).

In the model described here, only perceptual inputs—sensed rate of change in body configurations and other transitions—are controlled. Outputs vary in proportion to the discrepancy between reference and input. Given the anatomical connections of the BG, we hypothesize that the instantaneous position coordinates represented by SNr GABAergic output neurons are reference signals sent to the comparators in various position controllers. These signals are not sensory or motor. Such a classification is meaningless in a closed loop controller, because the output is not driven by sensory inputs or by descending commands; it is a result of a comparison between the two (**Figure 15**). The part of the sensory signal that deviates from the internal reference generates the appropriate amount of output, which acts through the feedback function in the environment.

In the control hierarchy only the lowest level has access to muscles. The higher order outputs from different controllers are used as descending signals that prescribe the desired amount of perceptual input for the immediately lower level. These are the orders sent to lower levels, not to command a specific output value, but to “request” a specific input value to be reached by the perceptual input functions of the lower level. The descending signals from the SNr GABAergic output neurons are not used to command muscle contractions, but to request specific values from specific position controllers in the tectum and pedunculopontine nucleus, which control orientation and body configuration.

### DA and gain control

We hypothesize that the sensorimotor striatum is organized as a topographically mapped modules, each dedicated to a specific velocity vector component corresponding to a movement direction (forward/backward, up/down, left/right). There are probably modules for different body parts, as different types of reference signals are generated for different controllers at lower levels (Carelli and West, 1991).

DA is hypothesized to serve as the gain in the transition controller. As a neuromodulator, DA does not directly cause firing of target neurons, though it can alter responsiveness of these neurons to inputs, e.g., glutamatergic corticostriatal inputs (Gerfen and Surmeier, 2011). Thus, DA can determine the magnitude of the velocity error signal, and the rate of change in body configurations.

**Figure 14:**
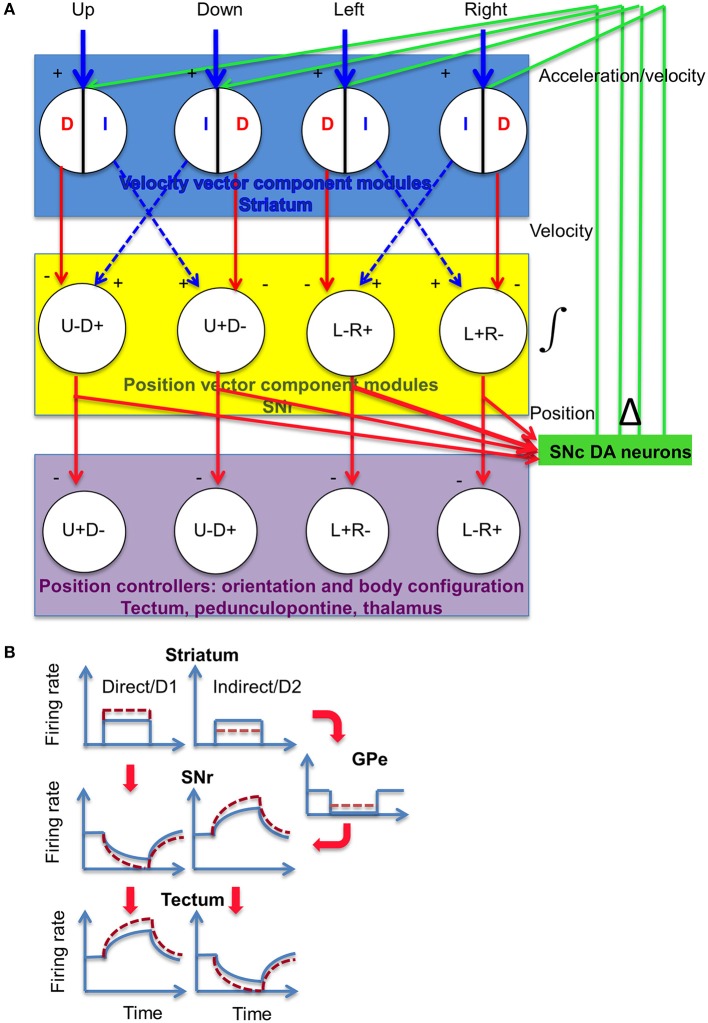
**Proposed model for DA modulation of striatal outputs. (A)** SNr neurons receive projections from the striatum and external globus pallidus, via the direct (D, striatonigral) and indirect (I, striatopallidal) pathways. The net effect on the SNr could be either inhibitory (minus sign) or excitatory (disinhibitory, plus sign). Both types of signals represent velocity error signals from the velocity controller. The dorsal striatum is hypothesized to contain at least four different modules, each responsible for movement in a specific direction. Striatal neurons can signal velocity error signals (Kim et al., 2014), which is integrated by the SNr and converted into position reference signal to position controllers in the midbrain and diencephalon (Barter et al., 2015). Using the outputs from the different modules, this circuit can perform vector addition to generate the actual movement vector. The magnitude of the signal entering the integrator is proportional to the rate of change in the integrator output. **(B)** Illustration of activity in the BG circuit, using a square pulse to represent a transient burst of action potentials with constant firing rate from striatal projection neurons. Dotted lines indicate altered firing rates as a result of DA modulation. DA is known to exert opposite effects on striatonigral neurons and striatopallidal neurons. Striatonigral neurons, which express D1 receptors, are increased by D1 activation, whereas striatopallidal neurons are inhibited by D2 activation(Gerfen and Surmeier, 2011). Moreover, activation of D1 receptors can also potentiate GABA release at the striatonigral terminals (Chuhma et al., 2011), whereas activation of D2 receptors can reduce GABA release at the pallidonigral terminals (Connelly et al., 2010; de Jesús Aceves et al., 2011). The net effect is consistent for the targets of SNr output. DA modulation has the net effect of potentiating the firing rate in a given position vector component, and further suppressing the antagonistic component. By increasing the rate of change in the position reference, the actual movement velocity is increased. As shown, both movement amplitude and speed are altered, but these variables can be independently controlled.

DA can have different effects on striatonigral (D1-expressing) and striatopallidal (D2-expressing) neurons giving rise to the direct and indirect pathways, respectively, but the ultimate effect is to modulate opponent output signals for downstream controllers (Figure 14). The organization of direct and indirect pathways implements a phase splitter that takes a common input and generates antiphase signals for commanding antagonistic controllers, e.g., opposite movement directions. The facilitating properties of the striatonigral synapse suggests that nigral integration of the inhibitory input is possible, whereas the depressing property of the pallidonigral synapse means that integration of the excitatory (disinhibitory) input is possible (Connelly et al., 2010; Zhou and Lee, 2011). On the other hand, the nigrotectal synapse appears to be neither facilitating nor depressing (Kaneda et al., 2008), so there does not appear to be a neural integrator in the tectum. This arrangement avoids having two integrators in the overall control hierarchy, which can create oscillations.

This hypothesis is supported by findings that neurons from both pathways are simultaneously activated during behavior (Cui et al., 2013; Isomura et al., 2013), and with opponent BG outputs from the SNr projection neurons (Fan et al., 2012; Rossi et al., 2013a; Barter et al., 2014, 2015).

### Relationship between BG output and DA activity

A major source of input is neighboring SNr GABA neurons, which inhibit DA neurons directly. These inhibitory projections come from collaterals of the GABAergic fibers which presumably synapse on other targets such as the tectum and ventral thalamus (Tepper and Lee, 2007). SNc DA neurons can fire tonically at a low rate by calcium entry through voltage-gated calcium channels; burst firing, on the other hand, appears to require NMDA currents produced by glutamatergic inputs. But due to tonic GABAergic inhibition from SNr outputs, reduced GABA release can also generate burst firing (Kang and Kitai, 1993; Tepper et al., 1995; Paladini et al., 1999). Local blockade of nigral GABA-A receptors causes bursting in DA neurons, which suggests tonic activation of GABA-A receptors on DA neurons. Thus, disinhibition is a major contributor to burst firing in DA neurons recorded here. Bursting is prevented by GABA-A inhibition even in the presence of glutamatergic inputs.

Whereas most DA neurons are correlated with velocity and acceleration, GABA neurons are correlated with instantaneous position coordinates (Barter et al., 2015). This pattern suggests that the DA neurons are in a position to take the derivative of the GABA outputs from the BG. Our results support this hypothesis (Figure 13), showing simultaneously recorded GABA and DA neurons in which the derivative of the GABA output is subtracted from the output of the DA neurons. Although these results are preliminary, they suggest specific pairing of GABA neurons and DA neurons that are classified by their preferred vector components.

A derivative of the BG output, then, can be fed back to the striatum. This could represent a mechanism for adaptive gain control, in which the gain can vary according to the movement velocity requirement. The gain of controllers for different body parts can also be independently adjusted. Haber and colleagues observed that the nigral output disinhibits a region of the SNc that send DA projections to the origin of the striatonigral projections as well as neighboring regions, forming a striatonigrostriatal loop (Haber et al., 2000). Currently it remains unclear how these projections are related to the functionally defined striatal modules shown in Figure 14. One possibility is that this type of velocity or acceleration feedback is sent to the same striatal neurons that generated the reference signal for movement in a particular direction, as well as neurons that generated the opponent signal. More generally, it will allow the rate of change in one controlled transition to adjust the gain for a different but related transition controller.

**Figure 15:**
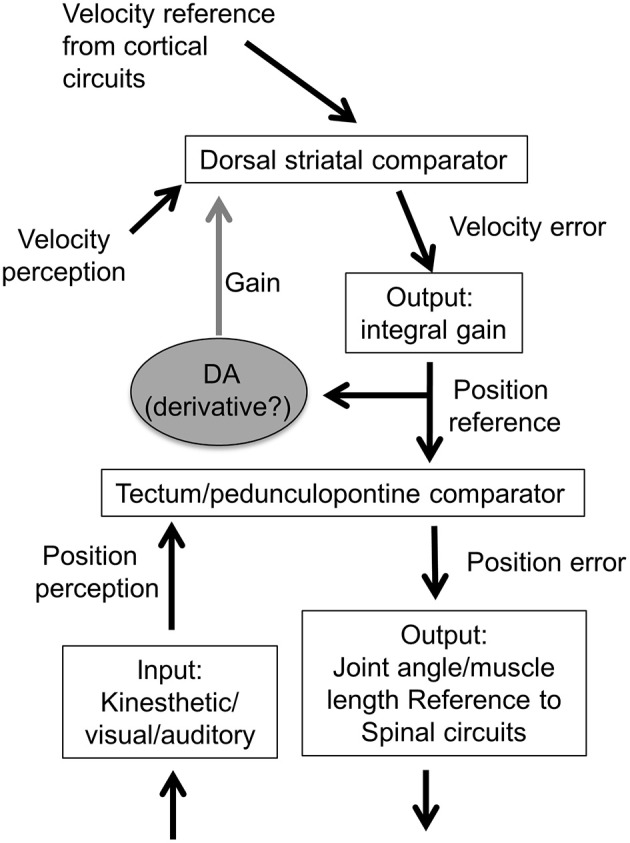
**Model of cascade control hierarchy for velocity and position control**. The velocity control system is hierarchically higher than the position control system. There are multiple position controllers, including those for orientation and body configuration, which can command hierarchically lower controllers for joint angle, muscle length, and muscle tension (not shown here). The lowest level is the tension or force controller, with alpha motor neurons acting as the comparator and muscles as the output function (Yin, 2014a).

On the other hand, because the SNr GABA output (position) is a time integral of the striatal output (velocity), the GABA neurons possess cellular properties that implement leaky integration of their inputs (Barter et al., 2015). This is integral gain in a transition control system, in which the error is a velocity signal, and the output represents the rate of change in position. In this cascade control arrangement, the higher “master loop” has an integrator, whereas the lower level position loop does not (Figure 15).

### Clinical implications

Because DA adjusts the gain of the velocity controller, it primarily affects the rate of change in perceptual variables, including those beyond the proprioceptive domain. This could explain common symptoms in various disorders—such as Parkinson's disease and Tourette syndrome—which are associated with abnormal DA levels. To put it simply, too much DA can result in faster movements as well as other types of perceptual transitions, whereas too little DA can result in a slowing down of the same transitions (Yin, 2014b). This could explain the continuum between hypokinetic and hyperkinetic disorders. On the other hand, the role of DA is to adjust the responsiveness of striatal neurons to glutamatergic inputs, so increasing glutamatergic signaling can have similar effects to a certain extent. In this circuit, the signal that matters is the spiking of the striatal neurons.

A reduction in DA reduces the magnitude of the velocity error signaled by the striatal projection neurons. Because there is a neural integrator in the SNr, the reduced rate of firing in striatal neurons results in reduced rate of change in body configurations. The total number of spikes from the striatal comparator can be reduced, so that movement amplitude is also reduced. In Parkinson's disease, which mainly involve degeneration of DA neurons in the SNc, both amplitude and speed of movements are reduced, which is why DeLong and colleagues proposed that BG output firing rate could encode both variables (DeLong, 1990). Although this hypothesis is wrong, the reduction in the gain of the velocity controller can result in reduced amplitude and speed of movements.

Another implication is suggested by the possible differentiation of the GABA output performed by DA neurons, as a result of the disinhibition mechanism discussed above. Such velocity or acceleration feedback is often used in engineering to prevent oscillations and overshoots, by damping the controller in motion control. The lack of such feedback may explain common symptoms in Parkinson's disease, which is characterized by tremor and neural oscillations (Brown, 2006; Costa et al., 2006).

One implication of our model is that, in principle, the contribution of the DA signal can be replaced. When DA signaling is insufficient, as in Parkinson's disease, it may be possible to take the derivative from the GABA projection neurons to generate the needed DA signal. The challenge is to find the appropriate place where this signal can be injected. Clearly the striatum is the main candidate, but too little is known about the organization of the functional striatal modules to identify the suitable place in the circuit where more gain can be added. Moreover, to enhance the gain of such a system requires recording neural activity and delivering stimulation in real time based on the recorded data. This could also be challenging, especially in clinical practice.

### Implications for motor control

In conventional studies attempting to relate neural activity with movement kinematics, the observed correlation between kinematic variables and single unit activity is very low (Paninski et al., 2004). When movement-related signals are used to move some load, e.g., in brain-machine-interface applications, extensive transformation of the neural data is needed. It is necessary to design a “decoder” to get the “control signal” needed to drive the effector (Taylor et al., 2002). Even though sensory feedback is often used, the feedback is not implemented correctly in the appropriate places in the control loop, due to the misunderstanding of inputs and outputs in the systems analysis. With this approach, there is no need for a neural signal, as any behavioral output can be transformed similarly, and even if neural signals are used, single unit activity is not necessarily better than population measures such as local field potential or electroencephalogram recording.

By contrast, we have shown a strikingly linear relationship between neural activity and kinematics. But this is not due to any coding of movement to be used by a decoder. Rather the signals recorded are the signals used in circuits that generate the movements. Strictly speaking, there is neither encoding nor decoding of movement kinematics.

### Rewards and other goals

The control of movement velocity is only one type of transition control. The controlled variable is the perceptual input representing the rate of change in kinesthetic variables, e.g., rate of change in joint angle (Mountcastle et al., 1975). To use this general purpose system, reference signals must enter the striatum, e.g., from the cerebral cortex.

There are multiple parallel cortico-BG networks (Alexander et al., 1986). Corticostriatal projections are topographically organized, with the sensorimotor striatum receiving inputs from primary motor and somatosensory cortices. According to our model, this sensorimotor network contains the velocity controller. The other networks would constitute different transition controllers with different controlled variables based on their sensory representations. The content of the signals they process can be very different, explain the well-known functional heterogeneity in the striatum (Yin et al., 2004, 2005, 2009). The controlled variables for the associative and limbic networks are not well-defined, but they are presumably related to exteroceptive and interoceptive inputs, respectively.

The term “reward” can be better defined as the control of some of these sensory representations that are sent to the striatum. The most immediate representation of the rate of food reward comes from controlled perceptual variables in behaviors like chewing and licking. The rate of change in these perceptual transitions can also be controlled in much the same way, though using a different set of effectors. Indeed, for orofacial consummatory behaviors, DA has been implicated in the control of reward rate (Rossi and Yin, 2015).

According to our model, what is often called “reward expectancy” can be viewed as an error signal in another higher-order reward rate controller. The reference for this controller is altered by changes in motivational state, i.e., satiety. Its error signal signals one more unit of the reward, just like marginal utility (Alchian and Allen, 1977), and commands the sensorimotor BG circuit by altering the reference value for movement velocity. Consequently, the actual velocity achieved is proportional to the higher order error signal. Thus, in food deprived animals, an increase in the size of the expected reward is known to associated with faster movements (Hikosaka, 2007). Of course, such a mechanism is not restricted to rewards, but would equally apply to a representation of impending danger.

## Conclusions

Given the known movement deficits following degeneration of DA neurons, it is hardly surprising that phasic DA activity can be related to movement kinematics. What is surprising is how long it took for this relationship to be uncovered. The failure of previous studies to identify the role of DA in shaping movement kinematics could be explained by the lack of continuous behavioral measurements in unrestrained animals (Romo and Schultz, 1990).

There is a fundamental difference between our approach and that taken in most previous studies. The traditional measure consists of discrete time stamps that are supposed to represent specific events labeled by the experimenter. This type of measurement creates the impression that behavior comprises a series of pulse-like events, and neural activity is supposed to be found around the time of these events. Although this approach may provide the appearance of rigor, it also makes it all too easy for the experimenter to ignore any behavior that is not described by the task labels, which often reflect his own perception of the experimental events and theoretical biases.

Perhaps there is a deeper reason for the popularity of conventional behavioral analysis in neuroscience (Yin, 2013). The dominant paradigm assumes that the nervous system converts inputs into outputs, through “sensorimotor transformation.” This open loop model of the organism leads to the convenient fiction of the passive animal processing information and generating motor responses and justifies the practice of heavily restraining animals to create the illusion of an input/output device. Instead, we assume behavior reflects the attempt to control specific sets of inputs in a hierarchy of control systems. Output varies to reach desired inputs, because the intrinsic reference signals dictate how much of the input is to be acquired. Behavioral output is not driven by sensory inputs, or by intrinsic signals in the absence of sensory input. It is always the result of a comparison between input and reference.

It has become customary in neuroscience to attribute behavioral variability to noise in sensory systems (Osborne et al., 2005). The underlying assumption, again, is that behavioral output is driven by sensory inputs. But in our model, behavioral variability has nothing to do with noise, but simply mirrors environmental disturbances to hidden controlled variables. In fact, although noise has always been a favorite concept that can be replied upon to explain away those undesirable features of behavior or neural activity (usually those features the experimenter fails to understand), from our perspective it is completely irrelevant. As shown by the present study, however variable the behavioral output may be, there is a linear relationship between the relevant neural signals and some time-varying measure of the continuous behavior. This relationship holds for any behavior, at any time, in any animal, with no need for averaging. As suggested by the present results, our approach promises to yield new insights into how neural activity generates behavior in the individual organism.

### Conflict of interest statement

The authors declare that the research was conducted in the absence of any commercial or financial relationships that could be construed as a potential conflict of interest.

## References

[B1] Alchian A. A. Allen W. R. (1977). Exchange and Production: Competition, Coordination, and Control. Belmont, CA: Wadsworth Publishing Company.

[B2] Alexander G. E. DeLong M. R. Strick P. L. (1986). Parallel organization of functionally segregated circuits linking basal ganglia and cortex. Annu. Rev. Neurosci. 9, 357–381. 10.1146/annurev.ne.09.030186.002041 3085570

[B3] Barter J. W. Castro S. Sukharnikova T. Rossi M. A. Yin H. H. (2014). The role of the substantia nigra in posture control. Eur. J. Neurosci. 39, 1465–1473. 10.1111/ejn.12540 24628921

[B4] Barter J. W. Li S. Sukharnikova T. Rossi M. A. Bartholomew R. A. Yin H. H. (2015). Basal ganglia outputs map instantaneous position coordinates during behavior. J. Neurosci. 35, 2703–2716. 10.1523/JNEUROSCI.3245-14.2015 25673860 PMC4323537

[B5] Boyden E. S. Zhang F. Bamberg E. Nagel G. Deisseroth K. (2005). Millisecond-timescale, genetically targeted optical control of neural activity. Nat. Neurosci. 8, 1263–1268. 10.1038/nn1525 16116447

[B6] Brown P. (2006). Bad oscillations in Parkinson's disease, in Parkinson's Disease and Related Disorders (Viena: Springer), 27–30.

[B7] Cagniard B. Beeler J. A. Britt J. P. McGehee D. S. Marinelli M. Zhuang X. (2006). Dopamine scales performance in the absence of new learning. Neuron 51, 541–547. 10.1016/j.neuron.2006.07.026 16950153

[B8] Cannon C. M. Palmiter R. D. (2003). Reward without dopamine. J. Neurosci. 23, 10827–10831. 14645475 10.1523/JNEUROSCI.23-34-10827.2003 PMC6740991

[B9] Carelli R. M. West M. O. (1991). Representation of the body by single neurons in the dorsolateral striatum of the awake, unrestrained rat. J. Comp. Neurol. 309, 231–249. 10.1002/cne.903090205 1885787

[B10] Chuhma N. Tanaka K. F. Hen R. Rayport S. (2011). Functional connectome of the striatal medium spiny neuron. J. Neurosci. 31, 1183–1192. 10.1523/JNEUROSCI.3833-10.2011 21273403 PMC3074638

[B11] Cohen J. Y. Haesler S. Vong L. Lowell B. B. Uchida N. (2012). Neuron-type-specific signals for reward and punishment in the ventral tegmental area. Nature 482, 85–88. 10.1038/nature10754 22258508 PMC3271183

[B12] Connelly W. M. Schulz J. M. Lees G. Reynolds J. N. (2010). Differential short-term plasticity at convergent inhibitory synapses to the substantia nigra pars reticulata. J. Neurosci. 30, 14854–14861. 10.1523/JNEUROSCI.3895-10.2010 21048144 PMC6633647

[B13] Costa R. M. Lin S. C. Sotnikova T. D. Cyr M. Gainetdinov R. R. Caron M. G.. (2006). Rapid alterations in corticostriatal ensemble coordination during acute dopamine-dependent motor dysfunction. Neuron 52, 359–369. 10.1016/j.neuron.2006.07.030 17046697

[B14] Cui G. Jun S. B. Jin X. Pham M. D. Vogel S. S. Lovinger D. M.. (2013). Concurrent activation of striatal direct and indirect pathways during action initiation. Nature 494, 238–242. 10.1038/nature11846 23354054 PMC4039389

[B15] de Jesús Aceves J. Rueda-Orozco P. E. Hernández R. Plata V. Ibañez-Sandoval O. Galarraga E.. (2011). Dopaminergic presynaptic modulation of nigral afferents: its role in the generation of recurrent bursting in substantia nigra pars reticulata neurons. Front. Syst. Neurosci. 5:6. 10.3389/fnsys.2011.00006 21347219 PMC3039203

[B16] DeLong M. R. (1990). Primate models of movement disorders of basal ganglia origin. Trends Neurosci. 13, 281–285. 10.1016/0166-2236(90)90110-V 1695404

[B76] Dommett E. Coizet V. Blaha C. D. Martindale J. Lefebvre V. Walton N.. (2005). How visual stimuli activate dopaminergic neurons at short latency. Science 307, 1476–1479. 10.1126/science.1107026 15746431

[B17] Fan D. Rich D. Holtzman T. Ruther P. Dalley J. W. Lopez A.. (2011). A wireless multi-channel recording system for freely behaving mice and rats. PLoS ONE 6:e22033. 10.1371/journal.pone.0022033 21765934 PMC3134473

[B18] Fan D. Rossi M. A. Yin H. H. (2012). Mechanisms of action selection and timing in substantia nigra neurons. J. Neurosci. 32, 5534–5548. 10.1523/JNEUROSCI.5924-11.2012 22514315 PMC6703499

[B19] Ferrier D. (1876). The Functions of the Brain. New York, NY: GP Putnam's Sons 10.1037/12860-000

[B20] Georgopoulos A. P. Schwartz A. B. Kettner R. E. (1986). Neuronal population coding of movement direction. Science 233, 1416–1419. 10.1126/science.3749885 3749885

[B21] Gerfen C. R. Surmeier D. J. (2011). Modulation of striatal projection systems by dopamine. Annu. Rev. Neurosci. 34, 441–466. 10.1146/annurev-neuro-061010-113641 21469956 PMC3487690

[B22] Grace A. A. Bunney B. S. (1984). The control of firing pattern in nigral dopamine neurons: single spike firing. J. Neurosci. 4, 2866–2876. 6150070 10.1523/JNEUROSCI.04-11-02866.1984 PMC6564731

[B23] Green A. M. Angelaki D. E. (2010). Internal models and neural computation in the vestibular system. Exp. Brain Res. 200, 197–222. 10.1007/s00221-009-2054-4 19937232 PMC2853943

[B24] Haber S. N. Fudge J. L. McFarland N. R. (2000). Striatonigrostriatal pathways in primates form an ascending spiral from the shell to the dorsolateral striatum. J. Neurosci. 20, 2369–2382. 10704511 10.1523/JNEUROSCI.20-06-02369.2000 PMC6772499

[B25] Hefti F. Melamed E. Sahakian B. J. Wurtman R. J. (1980). Circling behavior in rats with partial, unilateral nigro-striatal lesions: effect of amphetamine, apomorphine, and DOPA. Pharmacol. Biochem. Behav. 12, 185–188. 10.1016/0091-3057(80)90353-6 7189592

[B26] Hikosaka O. (2007). Basal ganglia mechanisms of reward-oriented eye movement. Ann. N.Y. Acad. Sci. 1104, 229–249. 10.1196/annals.1390.012 17360800

[B27] Isomura Y. Takekawa T. Harukuni R. Handa T. Aizawa H. Takada M.. (2013). Reward-modulated motor information in identified striatum neurons. J. Neurosci. 33, 10209–10220. 10.1523/JNEUROSCI.0381-13.2013 23785137 PMC6618603

[B28] Jin X. Costa R. M. (2010). Start/stop signals emerge in nigrostriatal circuits during sequence learning. Nature 466, 457–462. 10.1038/nature09263 20651684 PMC3477867

[B29] Kaneda K. Isa K. Yanagawa Y. Isa T. (2008). Nigral inhibition of GABAergic neurons in mouse superior colliculus. J. Neurosci. 28, 11071–11078. 10.1523/JNEUROSCI.3263-08.2008 18945914 PMC6671385

[B30] Kang Y. Kitai S. (1993). A whole cell patch-clamp study on the pacemaker potential in dopaminergic neurons of rat substantia nigra compacta. Neurosci. Res. 18, 209–221. 10.1016/0168-0102(93)90056-V 8127469

[B31] Kim N. Barter J. W. Sukharnikova T. Yin H. H. (2014). Striatal firing rate reflects head movement velocity. Eur. J. Neurosci. 40, 3481–3490. 10.1111/ejn.12722 25209171

[B32] Kravitz A. V. Freeze B. S. Parker P. R. Kay K. Thwin M. T. Deisseroth K.. (2010). Regulation of parkinsonian motor behaviours by optogenetic control of basal ganglia circuitry. Nature 466, 622–626. 10.1038/nature09159 20613723 PMC3552484

[B33] Lammel S. Steinberg E. E. Földy C. Wall N. R. Beier K. Luo L.. (2015). Diversity of transgenic mouse models for selective targeting of midbrain dopamine neurons. Neuron 85, 429–438. 10.1016/j.neuron.2014.12.036 25611513 PMC5037114

[B34] Leblois A. Wendel B. J. Perkel D. J. (2010). Striatal dopamine modulates basal ganglia output and regulates social context-dependent behavioral variability through D1 receptors. J. Neurosci. 30, 5730–5743. 10.1523/JNEUROSCI.5974-09.2010 20410125 PMC2866011

[B35] Lein E. S. Hawrylycz M. J. Ao N. Ayres M. Bensinger A. Bernard A.. (2006). Genome-wide atlas of gene expression in the adult mouse brain. Nature 445, 168–176. 10.1038/nature05453 17151600

[B36] Ljungberg T. Apicella P. Schultz W. (1992). Responses of monkey dopamine neurons during learning of behavioral reactions. J. Neurophysiol. 67, 145–163. 1552316 10.1152/jn.1992.67.1.145

[B37] Madisen L. Mao T. Koch H. Zhuo J. M. Berenyi A. Fujisawa S.. (2012). A toolbox of Cre-dependent optogenetic transgenic mice for light-induced activation and silencing. Nat. Neurosci. 15, 793–802. 10.1038/nn.3078 22446880 PMC3337962

[B38] Masino T. (1992). Brainstem control of orienting movements: intrinsic coordinate systems and underlying circuitry. Brain Behav. Evol. 40, 98–111. 10.1159/000113906 1422810

[B39] McClure S. M. Daw N. D. Read Montague P. (2003). A computational substrate for incentive salience. Trends Neurosci. 26, 423–428. 10.1016/S0166-2236(03)00177-2 12900173

[B40] Mountcastle V. B. Lynch J. Georgopoulos A. Sakata H. Acuna C. (1975). Posterior parietal association cortex of the monkey: command functions for operations within extrapersonal space. J. Neurophysiol. 38, 871–908. 808592 10.1152/jn.1975.38.4.871

[B41] Niv Y. Daw N. D. Joel D. Dayan P. (2007). Tonic dopamine: opportunity costs and the control of response vigor. Psychopharmacology 191, 507–520. 10.1007/s00213-006-0502-4 17031711

[B42] Osborne L. C. Lisberger S. G. Bialek W. (2005). A sensory source for motor variation. Nature 437, 412–416. 10.1038/nature03961 16163357 PMC2551316

[B43] Paladini C. A. Iribe Y. Tepper J. M. (1999). GABA A receptor stimulation blocks NMDA-induced bursting of dopaminergic neurons *in vitro* by decreasing input resistance. Brain Res. 832, 145–151. 10.1016/S0006-8993(99)01484-5 10375660

[B44] Paladini C. Roeper J. (2014). Generating bursts (and pauses) in the dopamine midbrain neurons. Neuroscience 282, 109–121. 10.1016/j.neuroscience.2014.07.032 25073045

[B45] Pan W.-X. Brown J. Dudman J. T. (2013). Neural signals of extinction in the inhibitory microcircuit of the ventral midbrain. Nat. Neurosci. 16, 71–78. 10.1038/nn.3283 23222913 PMC3563090

[B46] Paninski L. Fellows M. R. Hatsopoulos N. G. Donoghue J. P. (2004). Spatiotemporal tuning of motor cortical neurons for hand position and velocity. J. Neurophysiol. 91, 515–532. 10.1152/jn.00587.2002 13679402

[B47] Powers W. T. Clark R. K. McFarland R. L. (1960). A general feedback theory of human behavior. Percept. Mot. Skills 11, 71–88. 10.2466/pms.1960.11.1.71

[B48] Puryear C. B. Kim M. J. Mizumori S. J. (2010). Conjunctive encoding of movement and reward by ventral tegmental area neurons in the freely navigating rodent. Behav. Neurosci. 124, 234. 10.1037/a0018865 20364883 PMC2864532

[B49] Robinson D. (1981). The use of control systems analysis in the neurophysiology of eye movements. Annu. Rev. Neurosci. 4, 463–503. 10.1146/annurev.ne.04.030181.002335 7013640

[B50] Roesch M. R. Calu D. J. Schoenbaum G. (2007). Dopamine neurons encode the better option in rats deciding between differently delayed or sized rewards. Nat. Neurosci. 10, 1615–1624. 10.1038/nn2013 18026098 PMC2562672

[B51] Romo R. Schultz W. (1990). Dopamine neurons of the monkey midbrain: contingencies of responses to active touch during self-initiated arm movements. J. Neurophysiol. 63, 592–606. 2329363 10.1152/jn.1990.63.3.592

[B52] Rossi M. A. Fan D. Barter J. W. Yin H. H. (2013a). Bidirectional modulation of substantia nigra activity by motivational state. PLoS ONE 8:e71598. 10.1371/journal.pone.0071598 23936522 PMC3735640

[B53] Rossi M. A. Go V. Murphy T. Fu Q. Morizio J. Yin H. H. (2015). A wirelessly controlled implantable LED system for deep brain optogenetic stimulation. Front. Integr. Neurosci. 9:8. 10.3389/fnint.2015.00008 25713516 PMC4322607

[B54] Rossi M. A. Sukharnikova T. Hayrapetyan V. Y. Yang L. Yin H. H. (2013b). Operant self-stimulation of dopamine neurons in the substantia nigra. PLoS ONE 8:e65799. 10.1371/journal.pone.0065799 23755282 PMC3673941

[B55] Rossi M. A. Yin H. H. (2015). Elevated dopamine alters consummatory pattern generation and increases behavioral variability during learning. Front. Integr. Neurosci. 9:37. 10.3389/fnint.2015.00037 26029064 PMC4432675

[B56] Schultz W. (1998a). Predictive reward signal of dopamine neurons. J. Neurophysiol. 80, 1–27. 9658025 10.1152/jn.1998.80.1.1

[B57] Schultz W. (1998b). The phasic reward signal of primate dopamine neurons. Adv. Pharmacol. 42, 686–690. 10.1016/S1054-3589(08)60841-8 9327992

[B58] Schultz W. Dayan P. Montague P. R. (1997). A neural substrate of prediction and reward. Science 275, 1593–1599. 10.1126/science.275.5306.1593 9054347

[B59] Schultz W. Ruffieux A. Aebischer P. (1983). The activity of pars compacta neurons of the monkey substantia nigra in relation to motor activation. Exp. Brain Res. 51, 377–387. 10.1007/BF00237874

[B60] Sparta D. R. Stamatakis A. M. Phillips J. L. Hovelsø N. van Zessen R. Stuber G. D. (2012). Construction of implantable optical fibers for long-term optogenetic manipulation of neural circuits. Nat. Protoc. 7, 12–23. 10.1038/nprot.2011.413 22157972 PMC8647635

[B61] Taylor D. M. Tillery S. I. H. Schwartz A. B. (2002). Direct cortical control of 3D neuroprosthetic devices. Science 296, 1829–1832. 10.1126/science.1070291 12052948

[B62] Tepper J. Martin L. Anderson D. (1995). GABAA receptor-mediated inhibition of rat substantia nigra dopaminergic neurons by pars reticulata projection neurons. J. Neurosci. 15, 3092–3103. 7722648 10.1523/JNEUROSCI.15-04-03092.1995 PMC6577766

[B63] Tepper J. M. Lee C. R. (2007). GABAergic control of substantia nigra dopaminergic neurons. Prog. Brain Res. 160, 189–208. 10.1016/S0079-6123(06)60011-3 17499115

[B64] Todorov E. Jordan M. I. (2002). Optimal feedback control as a theory of motor coordination. Nat. Neurosci. 5, 1226–1235. 10.1038/nn963 12404008

[B65] Wang D. V. Tsien J. Z. (2011a). Convergent processing of both positive and negative motivational signals by the VTA dopamine neuronal populations. PLoS ONE 6:e17047. 10.1371/journal.pone.0017047 21347237 PMC3039659

[B66] Wang D. V. Tsien J. Z. (2011b). Conjunctive processing of locomotor signals by the ventral tegmental area neuronal population. PLoS ONE 6:e16528. 10.1371/journal.pone.0016528 21304590 PMC3029369

[B67] Wang S. Tan Y. Zhang J.-E. Luo M. (2013). Pharmacogenetic activation of midbrain dopamine neurons produces hyperactivity. Neurosci. Bull. 29, 1–8. 10.1007/s12264-013-1327-x 23516143 PMC5561950

[B68] White N. M. (1986). Control of sensorimotor function by dopaminergic nigrostriatal neurons: influence on eating and drinking. Neurosci. Biobehav. Rev. 10, 15–36. 10.1016/0149-7634(86)90030-8 3010199

[B69] Yin H. H. (2013). Restoring purpose in behavior, in Computational and Robotic Models of the Hierarchical Organization of Behavior (Berlin: Springer), 319–347.

[B70] Yin H. H. (2014a). How the basal ganglia output generates behavior. Adv. Neurosci. 2014:768313 10.1155/2014/768313

[B71] Yin H. H. (2014b). Action, time and the basal ganglia. Philos. Trans. R. Soc. Lond. B Biol. Sci. 369:20120473. 10.1098/rstb.2012.0473 24446506 PMC3895997

[B72] Yin H. H. Knowlton B. J. Balleine B. W. (2004). Lesions of dorsolateral striatum preserve outcome expectancy but disrupt habit formation in instrumental learning. Eur. J. Neurosci. 19, 181–189. 10.1111/j.1460-9568.2004.03095.x 14750976

[B73] Yin H. H. Mulcare S. P. Hilario M. R. Clouse E. Holloway T. Davis M. I.. (2009). Dynamic reorganization of striatal circuits during the acquisition and consolidation of a skill. Nat. Neurosci. 12, 333–341. 10.1038/nn.2261 19198605 PMC2774785

[B74] Yin H. H. Ostlund S. B. Knowlton B. J. Balleine B. W. (2005). The role of the dorsomedial striatum in instrumental conditioning. Eur. J. Neurosci. 22, 513–523. 10.1111/j.1460-9568.2005.04218.x 16045504

[B75] Zhou F. M. Lee C. R. (2011). Intrinsic and integrative properties of substantia nigra pars reticulata neurons. Neuroscience 198, 69–94. 10.1016/j.neuroscience.2011.07.061 21839148 PMC3221915

